# Knowledge and attitude of hepatitis B infection among patients with the infection in the main liver clinic in The Gambia

**DOI:** 10.11604/pamj.2022.42.252.34577

**Published:** 2022-08-05

**Authors:** Landing Jarju, Sheikh Omar Bittaye, Abdoulie Keita, Saydiba Tamba, Ramou Njie

**Affiliations:** 1 Department of Internal Medicine, School of Medicine and Allied Health Sciences, University of The Gambia, Banjul, The Gambia,; 2 Department of Internal Medicine, Edward Francis Small Teaching Hospital, Banjul, The Gambia,; 3 Department of Obstetrics and Gynaecology, Edward Francis Small Teaching Hospital, Banjul, The Gambia

**Keywords:** Knowledge, attitude, hepatitis B virus, patients, Gambia

## Abstract

**Introduction:**

adequate knowledge on hepatitis B virus (HBV) infection is important among patients with the infection as this impacts their health-seeking behavior. This study therefore assessed the knowledge and attitude among patients infected with HBV in The Gambia.

**Methods:**

this cross-sectional study was conducted at the main liver clinic, Medical Research Council Gambia at London School of Hygiene and Tropical Medicine (MRCG@LSHTM). A questionnaire was administered on a one-on-one basis to assess the level of knowledge and attitude of people with chronic HBV.

**Results:**

a total of 152 HBV patients were recruited into the study. Majority of the participants were male 136 (89.5%), within the 30-39 years age group. Sixty-four (42.1%) of the patients attained secondary education and 72 (47.4%) were working as civil servants. The mean knowledge score was 11.09/20 (standard deviation = 4.89). HBV patients having tertiary level education (p-value =0.001) or HBV diagnosis greater than 1 year (p-value =0.031) were more likely to have adequate knowledge of HBV infection. No significant associations were found between the socio-demographic and clinical characteristic variables and attitude. However, majority of the participants (56.6%) reported been worried about having HBV infection ever since being diagnosed.

**Conclusion:**

this study has highlighted the need for more patient health education especially for those with lower levels of education and newly diagnosed patients. It also further confirms the need for cultural and appropriate language consideration in providing education and information for HBV patients in The Gambia at the point of diagnosis.

## Introduction

Hepatitis B virus (HBV) infection is a major public health problem worldwide; roughly 30% of the world's population show serological evidence of current or past infection [1]. More than 2 billion people worldwide are estimated to have had hepatitis B virus infection with 350 - 400 million being chronic carriers of the virus [2,3]. The infection is the 10th leading cause of death resulting in 500 000 to 1.2 million deaths per year caused by chronic hepatitis, cirrhosis and hepatocellular carcinoma [2]. Although it is classified as a “disease of priority,” there is an incessant increase in the detection of new cases worldwide [4].

In Africa and Asia, it remains a major cause of morbidity and mortality, with prevalence rates higher than 8% [5]. There is a 15-25% risk of dying prematurely in adulthood from HBV-related cirrhosis and hepatocellular carcinoma, while a small proportion of those with acute infections may also succumb to fulminant liver failure [3].

In the Gambia, the rate of chronic hepatitis B virus infection carriage in the adult population is estimated at 8.2% [6]. Knowledge of HBV infection was extremely low in the communities and none of the HBsAg positive individuals had been previously tested and knew their status [7]. Despite its high prevalence and its positive association with liver cancer, no research has been done regarding the knowledge and attitude among patients with the infection. Since HBV infection is mostly a chronic infection that may end in devastating consequences, an increase level of understanding of the condition amongst those infected can reduce the transmission, improve treatment compliance and adherence and lessen the complications that may arise from the disease.

Knowledge, attitude and practice (KAP) surveys are representative of a specific population to collect information on what is known, believed and done in relation to a particular topic, and are the most frequently used study tool in health seeking behavior research [8]. The primary objective of this study is to assess the knowledge, attitude of chronic hepatitis B infection amongst patients with the infection in The Gambia.

## Methods

**Study design and population:** the study is a descriptive observational cross-sectional study conducted between August to November 2018. It was conducted at the main liver clinic at the Medical Research Council at London School of Hygiene and Tropical Medicine (MRCG@LSHTM) in Fajara. The clinic is the main referral center for all liver related conditions in the country and receives patients all through the length and breadth of the country.The clinic staff is comprised of field workers, cancer registrars, data entry managers, nurse specialists, laboratory technologists, trainee hepatologist (medical officers and residents) and is headed by a consultant gastroenterologist and hepatologist. Outpatient clinics are held four (4) days in a week. On average, 20-30 patients with positive hepatitis B serology are seen every day. The clinic also offers outreach services to the country´s main referral hospital, Edward Francis Small Teaching Hospital every Wednesday and a quarterly for rural hospital.

**Inclusion criteria:** any patient with Hepatitis B infection seen at the clinic.

**Exclusion criteria:** any Hepatitis B infection patient who declines to be part of the study and patients who are too ill to answer the questionnaire. Given that the prevalence of Hepatitis B among the Gambian population is 8.8% in community and 13% among prospective blood donors [7], the minimum sample size for simple proportion with 5% accuracy and 95% level of confidence is calculated to be 138.3 participants.

**Data collection and processing:** Hepatitis B positive patients who came into the liver clinic for routine medical consultation were approached and those who consented were recruited into the study using convenience sampling. A questionnaire was adopted from a similar study conducted in Malaysia [9] which looked at the “knowledge, Attitudes and Practices among People with Chronic Hepatitis B attending a hepatology clinic.” The questionnaire was pretested in 10 patients from the target population to help refine and clarify before been used for the actual study. It was written in English and was administered by the researcher and trained nurses working at the hepatology clinic of MRCG@LSHTM. All patients´ socio-demographic, clinical characteristics, time since diagnosis, knowledge and attitude of Hepatitis B were collected. Age and time since diagnosis were grouped. Knowledge section of the questionnaire was divided into sections assessing the participant´s general knowledge of hepatitis B virus infection, symptoms and signs of hepatitis B virus infection and mode of transmission. A scoring system was used to classify the participants as either having poor or adequate knowledge based on the calculated mean knowledge score of 11 out of the 20 item questions overall. A score of 11 or more is considered adequate, while that of less than 11 is considered poor.

The attitude section consists of 5 items and the responses are restricted to yes/no/don´t know. Responses with yes or don´t know were considered as a negative response while a no was considered as positive. Participants with 3 or more “no” responses were considered to have an overall positive attitude while those with less were considered to have an overall negative attitude.

**Ethical approval:** ethics approval for the study was granted by the Research and publication committee (RePubliC) of the University of The Gambia.

**Statistical analysis:** the data collected were entered into Statistical Package for Social Sciences (SPSS) and all analyses were made using SPSS. Descriptive statistics like frequencies and proportions were used to summarize the data. Chi square test at significance level of 0.05 where applicable and/or Fisher´s exact test at 95% confidence interval were used to determine significance. Phi and Cramer´s V were also used to determine the strength of the significance.

## Results

**Characteristics of the population:** a total of 152 HBV patients were recruited into the study and 2 patients were excluded because they decline to be part of the study. We also had 4 missing data from the ‘treatment status’, 3 from ‘occupation’, 1 from ‘time since diagnosis’ and 1 from the ‘point of diagnosis’ variables. Majority of the participants were male 136 (89.5%) with most of the participants within the 30-39 years age group 77 (50.7%). Sixty-four (42.1%) of the patients attained secondary level education and 42 (27.6%) had tertiary education. There were also 72 (47.4%) civil servants. Most of our participants were married 121(9.6%) and 50 (32.9%) were of the Mandinka tribe (Table 1). Sixty-one (40.1%) of the participants were diagnosed more than a year ago, 87 (57.2%) diagnosed at blood donation screening and only 20 (13.2%) were receiving treatment (Table 1).

**Table 1:** characteristics of the study population (N=152)

Socio-demographic variables		
Variables	Frequency (n)	Percentage (%)
Age		
Less than 30	38	25.0
30-39	77	50.7
40-49	33	21.7
50-59	2	1.3
Equal to or more than 60	2	1.3
Gender		
Male	136	89.5
Female	16	10.5
Level of education		
None	9	5.9
Madrassa (Arabic school)	21	13.8
Primary	16	10.5
Secondary	64	42.1
Tertiary	42	27.6
Occupation		
Unemployed	4	2.6
Student	8	5.3
Housewife	5	3.3
Petty trader	28	18.4
Artisan	33	21.7
Civil servants	72	47.4
Missing	3	2.0
Ethnicity		
Mandinka	50	32.9
Wolof	28	18.4
Fula	20	13.2
Jola	24	15.8
Serahulai	11	7.2
Others	19	12.5
Marital status		
Single	30	19.7
Divorced	1	0.7
Married	121	79.6
Time since diagnosis		
Less than or equal to 1 month	46	30.3
>1month-1 year	44	28.9
>1year	61	40.1
Missing	1	0.7
Point of diagnosis		
Blood donor screening	87	57.2
Community/clinic screening	64	42.1
Missing	1	0.7
Treatment status		
Yes	20	13.2
No	128	84.2
Missing	4	2.6
Attitude and Knowledge Level on Hepatitis B Infection		
Attitude		
Positive	107	70.4
Negative	45	29.6
Knowledge		
Adequate	83	54.6
Poor	69	45.4

**Knowledge about HBV:** the level of knowledge of hepatitis B was adequate in 83 (54.6%) of the patients (Table 1). The majority of the participants, 124 (81.6%), 103 (67.8%) and 82(53.9%) knew that hepatitis B virus infection can cause chronic inflammation of the liver, liver failure and liver cancer respectively. However, only 51 (33.6 %) of the participants knew that hepatitis infection is caused by the hepatitis B virus (Table 2). Most of the participants 118 (77.6%) knew that individuals infected with the virus can be asymptomatic. The most commonly known symptoms to the participants were abdominal distension 90 (59.2%) and tiredness 78 (51.3%). However, majority were not aware of jaundice, nausea/vomiting and abdominal pain as HBV infection symptoms (Table 2). Most of the participants knew the modes of transmission, with 136 (89.5%) asserting that HBV can be transmitted through blood transfusion, 102 (67.1%) through sharing of needles, 88 (57.9%) by contact with open wounds, 83 (54.6%) with regards to perinatal transmission, 81 (53.3%) by sexual intercourse and 80 (52.6%) by sharing personal items like toothbrushes and razors (Table 2).

**Table 2:** knowledge and attitude of patients on Hepatitis B infection (N=152)

Knowledge	Yes n (%)
**Hepatitis B is a**	
Bacterial infection	4(2.6)
Viral infection	51(33.6)
**Hepatitis B can cause**	
Chronic inflammation of the liver	124 (81.6)
Liver failure	103(67.8)
Liver cancer	82 (53.9)
**Hepatitis B symptoms**	
Asymptomatic	118 (77.6)
Jaundice	62 (40.8)
Tiredness	78 (51.3)
Nausea and vomiting	47 (30.9)
Abdominal pain	70 (46.1)
Abdominal distension	90 (59.2)
**Modes of transmission**	
Sexual intercourse	81 (53.3)
Perinatal transmission	83 (54.6)
Sharing needles	102 (67.1)
Blood transfusion	136 (89.5)
Contact with open wounds	88 (57.9)
Sharing personal items like toothbrushes	80 (52.6)
Coughing and sneezing	35 (23)
Casual contact	8 (5.3)
Sharing eating and drinking utensils	23 (15.1)
**Attitude**	
Worried of spreading HBV to family and friends	72(47.4)
Worried about having HBV infection since diagnosis	86 (56.6)
Embarrass to reveal HBV status	26 (17.1)
Patients with HBV die early	18 (11.8)
Unwilling to reveal HBV status to family and friends	18 (11.8)

**Predictors of knowledge level:** the results indicated that tertiary level of education (p=0.001) and HBV diagnosis more than one year (p-value =0.031) were more likely to have adequate knowledge of HBV infection (Table 3). However, the level of education (Cramer´s V = 0.357) was a stronger predictor of knowledge level while time since diagnosis (Cramer´s V = 0.214) was a moderate predictor of knowledge level.

**Table 3:** factors affecting level of knowledge (N=152)

	Poor Knowledge n=69(%)	Adequate Knowledge n=83(%)	P value
**Level of education**			
None	6 (6.7)	3 (3.6)	0.001
Madrassa	9(13)	12 (14.5)	
Primary	14 (20.3)	2 (2.4)	
Secondary	29 (42)	35 (42.2)	
Tertiary	11(15.9)	31 (37.3)	
**Time since diagnosis**			
≤ 1 month	28 (41.2)	18 (21.7)	0.031
1 month- 1 year	18 (26.5)	26 (31.3)	
> 1 year	22 (32.4)	39 (47)	

**Attitude about HBV:** over two-third of the participants 107 (70%) had a positive attitude towards hepatitis B infection while 45 (29.6%) had a negative attitude towards the infection (Table 1). More than half of the participants 86 (56.6%) were worried ever since diagnosed with hepatitis B virus infection. Less than half of the participants, 72 (47.4%) were worried of spreading the infection to family and friends and only 18 (11.8%) believe that people diagnosed with hepatitis B virus infection die in a short time. More than three quarters of the participants are not embarrassed to reveal their hepatitis B status and disclose their hepatitis B status to their close family members (Table 2).

**Predictors of attitude:** the study showed no significant predictor of attitude among the socio-demographic and clinical characteristics variables.

## Discussion

Hepatitis B virus infection remains to be a very important public health problem in The Gambia. KAP surveys have been important sources of data to design health intervention methods and public health policies that is representative of that population. In The Gambia, there is paucity of data regarding the knowledge and attitude of hepatitis B infection among patients with the infection. This study therefore helps to identify the gaps in the knowledge of HBV infection in patients with HBV infection in The Gambia.

The majority of our patients were young males, diagnosed during blood donation screening. These findings are similar to previous studies on blood donors [7]. Lesser number of participants were diagnosed during community or clinic screening which justifies the need for a nationwide screening and treatment program HBV infection in The Gambia.

This study showed that the knowledge level on Hepatitis B virus infection of majority of the patients diagnosed with hepatitis B virus infection is adequate. However compared to similar studies elsewhere, HBV patients in the Gambia were found to have poorer knowledge than those in Malaysia [9], Singapore [10] and Chinese Canadians [11]. This disparity in the level of knowledge may be explained in part by the marked differences in the literacy rate between the Gambia (55.5%), Malaysia (94.6%) and Singapore (97%). The different methods of knowledge measurement across these studies/countries could have also contributed to the observed differences. Provider-related factors should also be considered in relation to study participant´s low overall knowledge scores. Previous studies have reported that patient´s inability to understand medical jargon, their fear of asking a medical practitioner for explanations about terminology, inadequate consultation time to address all queries due to over-burdened schedules, and their anxiety about and fear of the disease are major barriers to effective communication between patients and healthcare staff [12]. These suggest that equipping providers with effective communication skills as part of comprehensive disease prevention programs will help improve patient´s knowledge in our Hepatitis clinics.

The study identified tertiary level of education and HBV diagnosis of more than one year as significant predictors of higher knowledge scores. Participants with tertiary level education have higher levels of literacy, mostly able to search information regarding the infection online and are able to easily understand the information given to them by their healthcare providers. Other studies have shown that higher educational level attainment is associated with better HBV knowledge [10, 13, 14]. The significant relationship between higher level of knowledge of Hepatitis B infection and HBV diagnosis of more than one year may have also been due to the repeated education sessions and contacts with trained health personnel. Having lived with HBV infection and in regular contact with healthcare professionals enable the patients to seek more information with regard to HBV [9]. This maybe different from newly diagnosed patients who may have had one or no consultation. The comprehensive disease prevention program should therefore also include a robust Hepatitis B health education package in the first visit or once a patient is found to be positive.

More than half of the participants in this study were aware of the three significant complications of chronic hepatitis B virus infection namely chronic liver disease, liver cirrhosis and hepatocellular carcinoma. However, only one third of the participants knew that hepatitis B infection is caused by a virus. The possible reason for this is the difficulty in finding a fitting translation of hepatitis B in our local languages. Since the literacy rate in The Gambia is low coupled with difficulty in understanding or translating medical jargons, communication between the health workers and the patients will definitely be impaired. Our findings confirms that understanding of hepatitis B may be influenced by cultural background, and that culturally and linguistically appropriate interventions are required to improve knowledge and assist in providing higher standards of care [15].

The study also showed that over two-thirds of the participants knew that infection with hepatitis B virus can be asymptomatic. However, other studies have shown that many HBV patients did not know chronic HBV can be asymptomatic and may pass on the virus [16], and even would remain asymptomatic when the disease became much severe or in early disease such as liver cancer [10, 11]. Sometimes the symptoms may be mistaken for other minor medical health conditions such as indigestion and are usually treated with over-the-counter medication. This is of concern as those who experience these symptoms may not be aware of the severity of the condition and thus may cause delay in seeking treatment. Early detection of the complication of HBV is important so that appropriate treatment can be given before the disease progresses. Patients should also be made aware that cirrhosis and liver cancer can also be asymptomatic [9]. In addition, more than half of the participants were able to correctly respond to tiredness and abdominal distension as symptoms of HBV infection but majority were less aware of jaundice, nausea/vomiting and abdominal pain. These findings are similar to the Malaysian study [9].

The misperception that hepatitis B virus infection can be transmitted by sharing, eating and drinking utensils is very low among the patients in this study. However, this misperception seem to prevail among Asian HBV patients [10, 11, 17] and the general public in Singapore, San Francisco, Washington and Australia [18-20].The absence of these misperception could therefore reduce the anxiety in individuals diagnosed with the infection and make it easier for them to engage in open discussion regarding their HBV status with their family friends.

The majority of the participants in the study were worried about having HBV infection ever since being diagnosed. Been aware of the negative consequences of Chronic Hepatitis B infection may be a factor. Some other studies have also reported a positive association between anxiety and knowledge of other disease/conditions including inflammatory bowel disease, cardio vascular diseases and performing colposcopy. While anxiety can support health information seeking for some people, it can be counter-productive in others [21]. This further justifies a robust Hepatitis B health education package in the first visit or once a patient is found to be positive.

Most of the participants also showed a positive attitude towards disclosing their status and majority were not embarrassed to reveal their HBV status. The reason could be that disclosing their status especially to their family members could stop the spread of the infection to the family and friends. By informing their family members of the disease status, steps can also be taken by their family members to go for screening which could lead to early diagnosis and treatment or vaccination for prevention [9]. A qualitative study has also revealed that fear of rejection and stigma at a personal and community level is a barrier for those with HBV to disclose their disease status as the revelation would lead to segregation from family and friends [15], which is not the case in this study.

Less than half of the participants were worried of spreading the infection to family and friends. The reason for this could be due to the fact that the level of misperception of hepatitis B virus infection transmission by sharing, eating and drinking utensils is very low among our patients. Furthermore, the level of knowledge regarding different modes of transmission of HBV infection (Sexual intercourse, perinatal transmission, blood transfusion and sharing needles) was also high in our study.

The majority of our participants were not worried about the effect of HBV infection on their life span. The religious belief that anything that man has is the result of destiny and life span of any individual is only and solely determined by God maybe the reason for this.

This study was carried out in a single health facility. Even though, it is the main liver clinic in The Gambia it may not represent the full picture of hepatitis B infected patients in The Gambia. Besides, the study could also be subject to sample selection bias. Furthermore, it did not also cover all possible areas of knowledge and attitude of hepatitis B virus infection. Despite these limitations, the study has given us an insight about the knowledge and attitude of HBV infection among the infected patients in The Gambia.

## Conclusion

This study has highlighted the need for more patient education on HBV infection, especially for those with lower levels of education and newly diagnosed patients. It also further confirms the need for cultural and appropriate language consideration in providing education and information for HBV patients in The Gambia at the point of diagnosis.

### What is known about this topic

- *Increase level of knowledge of Hepatitis B patients will help reduce transmission, improve treatment compliance, adherence and lessen the complications that may arise from the disease;*
- *Knowledge of HBV infection is extremely low in the communities in The Gambia*.

### What this study adds

- *The knowledge level on Hepatitis B virus infection of majority of the patients diagnosed with HBV is adequate but low compared to other developed countries possibly due to the low literacy rate in The Gambia;*
- *HBV infected patients with tertiary level of education and HBV diagnosis of more than one year are more likely to have adequate knowledge of HBV infection in The Gambia;*
- *The majority of the participants in this study were worried about having HBV infection ever since being diagnosed*.
